# Supplementary material for: A survey of RNA secondary structural propensity encoded within human herpesvirus genomes: global comparisons and local motifs
Source: PeerJ. 2020 Sep 10;8:e9882. doi: 10.7717/peerj.9882 (PMC7487152; doi:10.7717/peerj.9882)
Supplement: Supplemental Information 2 [file peerj-08-9882-s002.pdf]

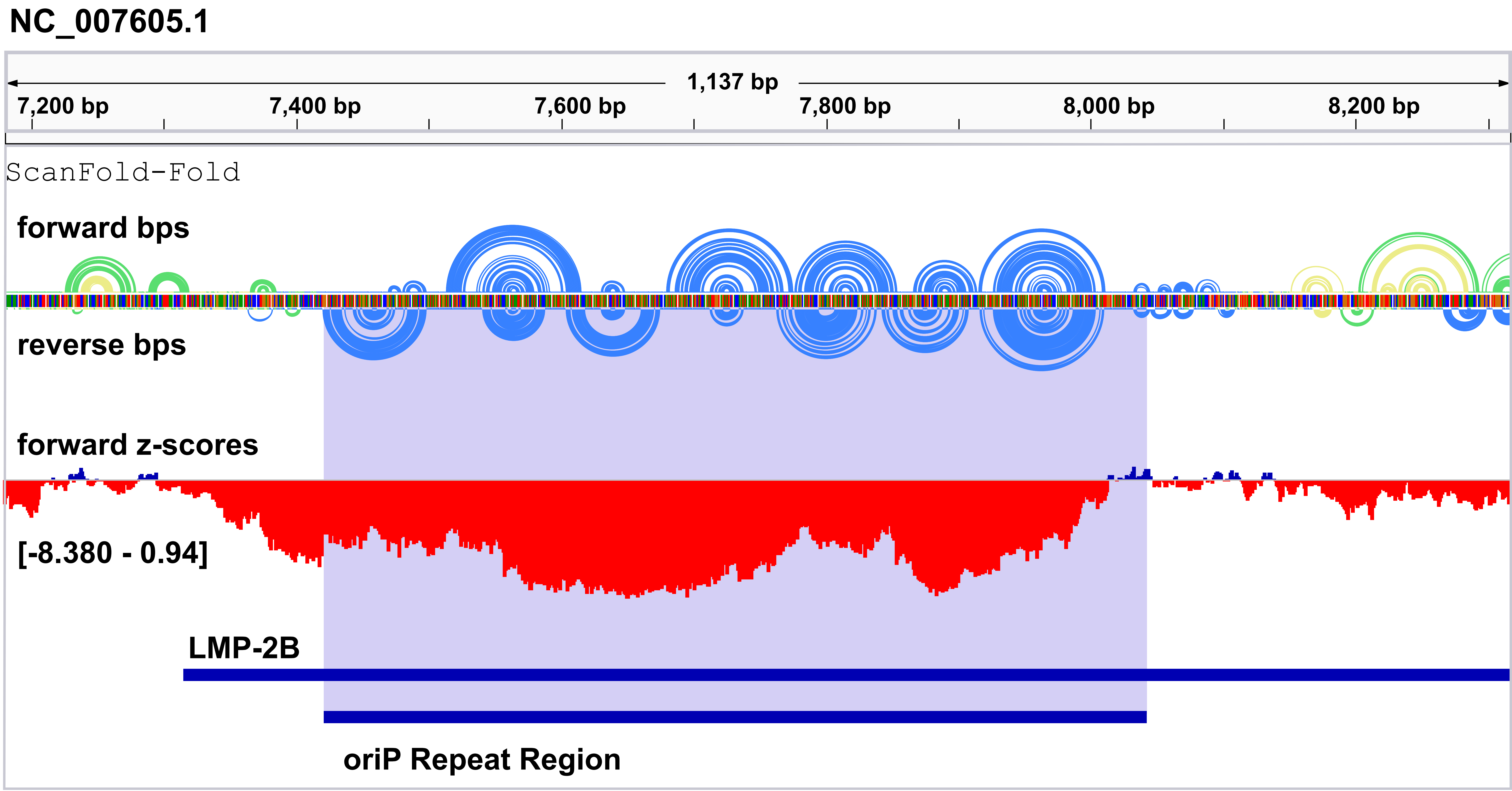

Supplemental Figure 2:Results for the oriP Region of the EBV genome. Shown is an IGV visualization of results. EBV-1 genome coordinates are followed by ScanFold-Fold predicted base pairs for both the forward and reverse sequences are represented as arcs (colored blue and green for bp with average z-score < -2 and -1, respectively, and yellow for bp with negative average z-scores > -1), and the genome sequence (A, C, G, and T are in green, blue, orange and red, respectively). Below this is a bar-graph showing the forward window z-score values predicted by ScanFold-Scan (each bar sits at the 1st nt of the window and is colored red or blue for negative/positive values, respectively; the range of values are indicated in brackets).
