# Supplementary material for: A survey of RNA secondary structural propensity encoded within human herpesvirus genomes: global comparisons and local motifs
Source: PeerJ. 2020 Sep 10;8:e9882. doi: 10.7717/peerj.9882 (PMC7487152; doi:10.7717/peerj.9882)
Supplement: Supplemental Information 8 — These values were used to generate the bar/line graph in Fig. 6B. [file peerj-08-9882-s008.docx]

|  | Control | WT | M1-2 | M3-6 | M7-8 | Description of terms |
| --- | --- | --- | --- | --- | --- | --- |
| FF RLU | 85304.67 | 93904.00 | 71941.00 | 62088.33 | 92879.00 | FireFly Relative Light Units, a measure of luciferase protein abundance |
| RL RLU | 81188.00 | 55505.33 | 152909.67 | 52691.00 | 27158.33 | Renilla Relative Light Units, a measure of luciferase protein abundance |
| RRR | 0.94 | 0.59 | 2.07 | 0.84 | 0.29 | Relative Response Ratio=(RL RLU)/(FF RLU), an assay normalization |
| S.D. RRR | 0.09 | 0.04 | 0.12 | 0.07 | 0.01 | Standard Deviation of RRR |
| RRR (%) | 100.00 | 62.61 | 219.52 | 89.63 | 30.76 | Percent of each condition normalized to “Control” which is set to 100% |
| S.D. | 14.02 | 7.48 | 25.23 | 11.77 | 3.43 | Standard Deviation of RRR% |
| t-test (RRR%) | - | **0.004** | **0.0002** | 0.154 | **0.0003** | **T-test correlations of RRR experimental conditions to the control** |
| 2^-ΔΔCt^ | 1.00 | 0.09 | 0.06 | 2.16 | 1.15 | A qPCR analysis metric, a measure of RL mRNA relative to FF mRNA. |
| S.D. | 0.16 | 0.02 | 0.04 | 0.20 | 0.20 | Standard Deviation of 2^-ΔΔCt^ |
| TE | 0.96 | 6.97 | 35.46 | 0.39 | 0.26 | Translational Efficiency=RRR/2^-ΔΔCt^, for a given condition |
| S.D. | 0.21 | 1.73 | 11.58 | 0.04 | 0.04 | Standard Deviation of TE |
| TE (%) | 100.00 | 726.73 | 3694.89 | 40.81 | 26.95 | TE expressed as a percent relative to the Control, which is normalized to 100 |
| S.D. | 31.36 | 242.04 | 1458.27 | 10.13 | 7.59 | Standard Deviation of TE (%) |
| t-test (TE) | - | **0.004** | **0.007** | **0.010** | **0.005** | **T-test correlations of TE experimental conditions to the control** |

**Table S1.** Results of dual luciferase assays and RT-qPCR for motifs in the overlapping BFRF1-3 region when inserted into the pIS2 vector 3'UTR. The p-values for two sample t-tests (of TE or RRR% versus control) have been performed assuming unequal variance, with bolded values showing significance of p <0.05.
