# Supplementary material for: A survey of RNA secondary structural propensity encoded within human herpesvirus genomes: global comparisons and local motifs
Source: PeerJ. 2020 Sep 10;8:e9882. doi: 10.7717/peerj.9882 (PMC7487152; doi:10.7717/peerj.9882)
Supplement: Supplemental Information 1 [file peerj-08-9882-s001.pdf]

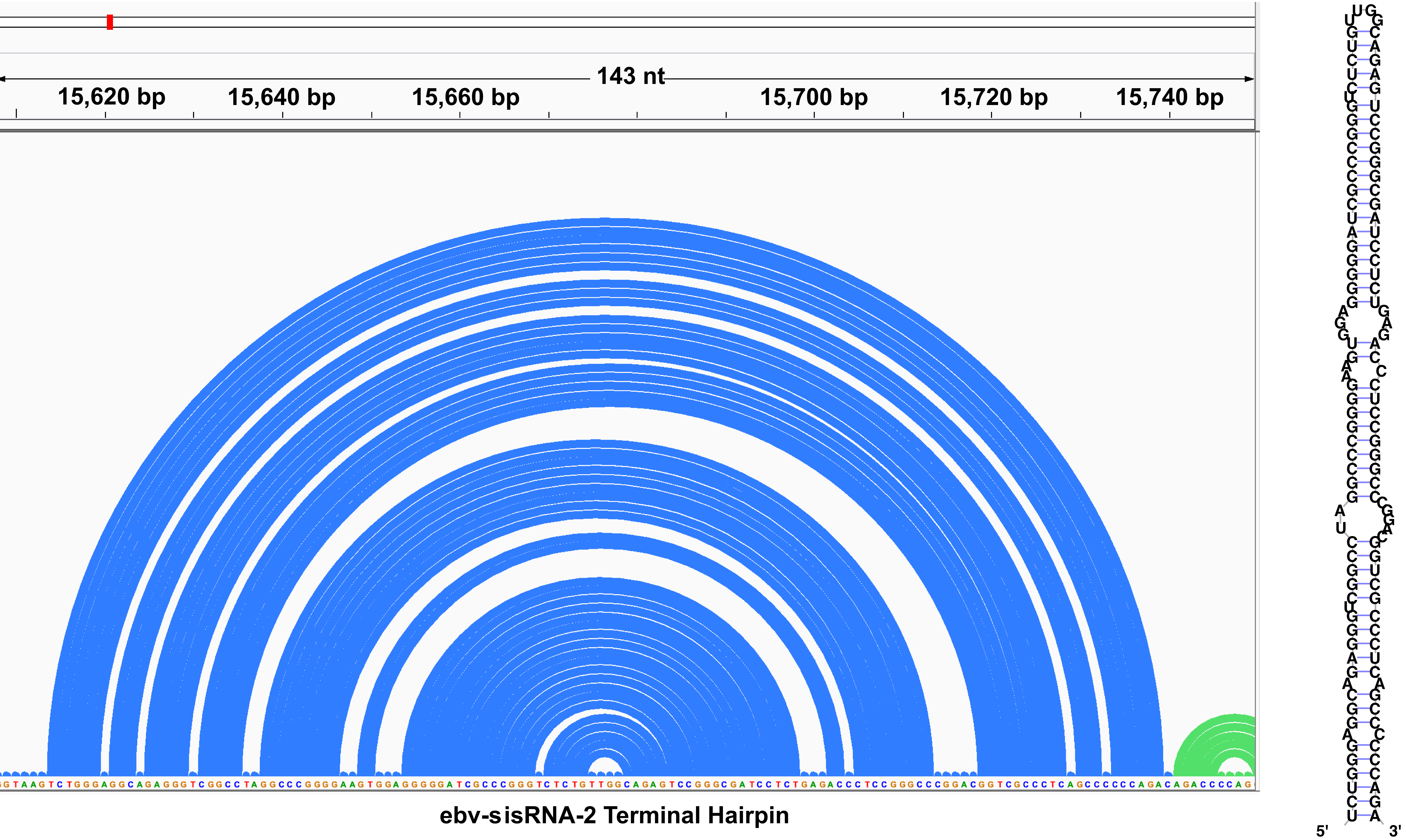

Supplemental Figure 1: Results for the ebv-sisRNA-2 Terminal Hairpin. Here is an IGV visualization of results. EBV-1 genome coordinates are followed by ScanFold-Fold predicted base pairs represented as arcs (colored blue and green for bp with average z-score < -2 and -1, respectively) and the genome sequence (A, C, G, and T are in green, blue, orange and red, respectively). A 2-D representation of the ScanFold predicted bp of ebv-sisRNA-2 Terminal Hairpin is shown on the right.
